# Improved Bone Regeneration Using Biodegradable Polybutylene Succinate Artificial Scaffold with BMP-2 Protein in a Rabbit Model

**DOI:** 10.3390/ma18102234

**Published:** 2025-05-12

**Authors:** Giulio Edoardo Vigni, Mariano Licciardi, Lorenzo D’itri, Francesca Terracina, Sergio Scirè, Giuseppe Arrabito, Bruno Pignataro, Lawrence Camarda, Giovanni Cassata, Roberto Puleio, Lucio Di Silvestre, Luca Cicero

**Affiliations:** 1 Department of Orthopaedics and Traumatology, University of Palermo, 90133 Palermo, Italy; giulio.vigni@gmail.com (G.E.V.); dr.ditri@gmail.com (L.D.); lawrence.camarda@unipa.it (L.C.); 2 Department of Biological, Chemical, and Pharmaceutical Sciences and Technologies (STEBICEF), University of Palermo, Via Archirafi 30, 90132 Palermo, Italy; francesca.terracina@unipa.it (F.T.); sergio.scire@unipa.it (S.S.); 3 Department of Physics and Chemistry “Emilio Segrè”, University of Palermo, Viale delle Scienze, Edificio 17, 90128 Palermo, Italy; giuseppedomenico.arrabito@unipa.it (G.A.); bruno.pignataro@unipa.it (B.P.); 4 Centro Mediterraneo Ricerca e Training (Ce.Me.Ri.T), Istituto Zooprofilattico Sperimentale della Sicilia “A. Mirri”, 90129 Palermo, Italy; giovanni.cassata@izssicilia.it (G.C.); luca.cicero@izsicilia.it (L.C.); 5 Laboratorio Istopatologia e Immunoistochimica, Dipartimento Ricerca Biotecnologica e Diagnostica Specialistica, Istituto Zooprofilattico Sperimentale della Sicilia “A. Mirri”, 90129 Palermo, Italy; roberto.puleio@izssicilia.it; 6 Department of Health Promotion, Mother and Child Care, Internal Medicine and Medical Specialties “G D’Alessandro”, University of Palermo, 90133 Palermo, Italy; lucio.disilvestre@unipa.it

**Keywords:** polybutylene succinate, microfibrillar scaffold, rabbit, bone reconstruction, bone regeneration, bone defect, BMP-2

## Abstract

Extensive bone loss represents a great challenge for orthopedic and reconstructive surgery. On an in vivo rabbit model, the healing of two bone defects on a long bone, tibia, was studied. A polybutylene succinate (PBS) microfibrillar scaffold was implemented with BMP-2 protein and hydroxyapatite (HA) as potential osteogenic factors. The present study was carried out on 6 male New Zealand white (4–6 months old) rabbits in vivo model. One bone defect was created in each subject on the tibia. The controls were left to heal spontaneously while the study samples were treated with the polybutylene succinate (PBS) microfibrillar scaffolds doped with BMP-2 and HA. Histological and immunohistochemical analyses were performed after euthanasia at 3 and 6 months. The bone defect treated with the BMP-2 PBS scaffold shows, from 3 months, a significantly increased presence of activated osteoblasts with mineralized bone tissue deposition. This study confirms the great potential of PBS scaffolds in the clinical treatment of bone defects.

## 1. Introduction

Despite its high healing potential, spontaneous healing of major bone defects, such as those following tumour resection, infection or severe trauma, is almost never possible. Although allogeneic and xenogeneic grafts have been used for these purposes, the gold standard is autogenous transplantation, especially from the iliac bones [1]. Tissue engineering has found three-dimensional scaffolds to be one of the most promising resources for bone tissue regeneration. These biomaterials must have specific characteristics, such as high mechanical strength or physical properties suited to the form and function of the bone structure into which they are to be implanted [2]. Not all biocompatible materials are applicable to bone defect as demonstrated by Lim et al. with a scaffold that prevented bone regeneration [3]. On the other hand, materials that are already widely used in bone related human surgery, like hydroxyapatite, proved to be biocompatible [4]. To date, the ideal scaffold has not yet been realized [5], so scientific research is also focused on engineering the available ones. The aim is to enhance the effect of scaffolds and at the same time counteract the specific limitations of certain materials. For example, Ma et al. studied the effect of scaffold on the tibia in rabbits model trying to counteract the lack of angiogenic properties and undesirable mechanical properties, such as brittleness, of hydroxyapatite. Therefore, they created a polyvinyl-alcohol/collagen/nanoattapulgite/hydroxyapatite composite scaffold, and, with the synergistic effects of these elements, they were able to demonstrate the promotion of vascularization and bone formation [6]. In 2024, based on the same assumptions and again with the intention of induced angiogenesis, Lin et al. studied a novel 3D-printed hydroxyapatite/carboxymethyl chitosan/polydopamine/total flavonoids of Rhizoma Drynariae scaffold [7]. In 2018, a Chinese research group [8], on a rabbit animal model with radial defect, demonstrated that an enhanced osteogenic effect can be achieved, using an engineered scaffold with osteoinductive periosteum mimetic. In 2020, Teotia et al. [9], compared in vivo in the rabbit the use of 3D-printed porous composite scaffolds with resins and osteoinductive growth factors in critical bone defects on flat (skull) and long bones (tibia). The tibia study was smaller in number than the skull study, but compared to the control group without scaffolds, faster healing of bone defects was demonstrated in both districts. Thus, each scaffold model requires careful studies to determine its biocompatibility and biodegradability. Scaffold-based tissue engineering must also take into account both pore size and mechanical properties: an increase in pore size is generally accompanied by a decrease in mechanical strength [2]. In this field Polybutylene succinate (PBS) scaffold has proven to be an excellent biomaterial in various fields, with high biocompatibility and easy processability by electrospinning technique [10,11,12,13,14]. Recently, a PBS microfibrillar scaffold showed to be an effective technique in achieving enhanced bone regeneration in critical bone defect [13]. The absence of adverse events demonstrates the biocompatibility and biodegradability properties of the scaffold. CT scans and histologic evaluation demonstrated the qualitative and quantitative improvement in bone regeneration compared with spontaneous healing in a calvarial defect. To date, no PBS scaffold has ever been studied for bone defects on the tibia in a rabbit animal model. Pförringer et al. [15] in 2018, implanted calcium sulphate and antibiotic scaffolds into the tibial metaphysis of rabbits, demonstrating excellent biocompatibility and ability to improve mechanical stability. Nowadays it’s essential that the scaffold is able to provide not only mechanical properties but also has the capabilities to stimulate bone growth with molecules previously loaded into the scaffold [16]. In 2017, a review of Kim et al. [17], showed that incorporated osteoinductive and osteoconductive materials, such as calcium and phosphate, into 3D scaffolds stimulate osteogenic differentiation of stem cells. Furthermore, it has been shown that increasing the local concentration of phosphate ion in the scaffolds promotes the activation of transcription factors, such as osteocalcin and osteopontin, which induce osteogenic differentiation in stem cells [17]. The same result was obtained using biodegradable polymeric scaffolds composed of calcium phosphate and bone formation-related growth factors (Bone morphogenetic protein-2 (BMP-2) and TGF-β3). In vitro studies have shown that BMP-2 increases angiogenesis in fracture healing by stimulating the expression of differentiation markers (osteocalcin, alkaline phosphatase), mineralised bone nodules [18,19] and VEGF secretion in osteoblasts [20,21]. Therefore, BMP-2 is a growth factor with osteoinductive properties and has been shown to promote the differentiation of stem cells into bone tissue [20].

BMP-2 has a half-life of between 7 and 16 min, so it needs to be applied in high doses to achieve clinical efficacy [22]. However, the use of supraphysiological doses of BMP-2 has been associated with adverse effects, including heterotopic bone formation and inflammation [23]. Therefore, a device that allows a gradual release of BMP-2 is needed to minimise these side effects. For this purpose, bioceramics, such as hydroxyapatite or beta-TCP, or materials such as collagen membranes have been used [24,25,26]. Kalay et al. in 2022 studied bone defects of the tibia with distraction osteogenesis [27]. After tibial osteotomy they placed an external fixator, increasing the distraction by 0.6 mm every day for 26 days. Control and engineered groups with BMP-2 and deferoxamine (DFO) were compared. The increase in VEGF activity, given by the growth factors, stimulated angiogenesis efficiently but without showing significant differences with the control group. Therefore, even though the BMP-2 group proved to be the one with the highest load-to-failure, the results showed that the mechanical stability of the external fixator is the fundamental condition for bone healing. Therefore, growth factors in a biomechanically unfavourable condition may not be sufficient for favourable bone regeneration. This study therefore confirms that the mechanical support of the scaffold element is crucial. The effects of scaffolds incorporating BMP-2 have been extensively studied in preclinical and clinical studies, especially on collagen-based scaffolds [28,29,30]. However, there is still room for improving therapeutic results and finding the most suitable scaffold combination [28,29,30].

The main aim of this study is to demonstrate, in an in vivo rabbit model, the ability to increase bone regeneration in a long bone subject to weight bearing stress, upon implantation of a new electrospun microfibrillar polybutylene succinate scaffold, loaded with osteogenic factors, such as the bone formation-related growth factors BMP-2 and hydroxyapatite.

## 2. Materials and Methods

### 2.1. Scaffold Preparation and Characterization

PBS scaffold was produced following a procedure already published [14]. Poly 1,4-butylene succinate extended with 1,6-diisocyanatohexane (Sigma-Aldrich, Gillingham, UK) solution (15% *w*/*v* in 1,1,1,3,3,3-hexafluoroisopropanol) was mixed with hydroxyapatite powder (HA, 10% *w*/*w* respect to PBS weight, 75 mg) and the antibacterial molecule ciprofloxacine (CPX, 5% *w*/*w* respect to PBS, 37.5 mg) used as antimicrobial preservant to prepare the starting dispersion for electrospinning. The electrospinning process was carried out vertically with 15 kV voltage using a NF 103 Electrospinning (MECC, Fukuoka, Japan) and a constant polymeric solution rate (0.8 mL/min) obtained through a programmable syringe pump. The electrospun scaffold was collected on a stainless-steel plate positioned 15 cm below the tip of the needle.

Morphological characteristics of scaffolds were investigated with a scanning electron microscope (ESEM Philips XL30, Milan, Italy) operating at 5 kV. Each sample was deposited onto a carbon-coated steel stub, dried under vacuum (0.1 Torr), and sputter-coated with gold (15 nm thickness) prior to microscopy examination.

3D structure of the scaffold was analyzed with a μCT scanner (Skyscan 1272, Bruker Kontich, Belgium) at a source voltage of 40 kV, a current of 250 mA, a total rotation of 180° and a rotation step of 0.3°. No filter mode was chosen for the acquisitions. The image pixel size was 2.6 μm and the scan duration was about 3 h for every sample. The scanning dataset obtained after the acquisition step consisted of images in 16-bit tiff format (3238 × 4904 pixels). The 3D reconstructions were carried out using the software NRecon (version 1.6.10.2) starting from the acquired projection images. The obtained 2D-images had a color depth of 8 bit with 265 grey levels. The whole set of raw images were displayed in a 3D space by the software CTVox.

### 2.2. Loading of Bone Morphogenetic Protein (BMP2)

Bone morphogenetic protein 2 (recombinant human BMP-2, 10 mcg, Merk) was loaded onto the scaffold using two different procedure: a printing deposition procedure and a drop adsorption procedure. The protein loading procedure by impregnation was instead carried out by depositing the protein solution, drop by drop, directly on the surface of the microporous scaffold and subsequently dehydrating under sterile box for 24 h. To print bone morphogenetic protein 2 (recombinant human BMP-2, 10 mcg, Merk) an aqueous solution at the concentration of 5 mcg/mL, was by using a precision printer, Dimatix DMP 2800 Fujifilm (Fujifilm Dimatix Inc., Santa Clara, CA, USA), equipped with piezoelectric nozzles capable of dispensing droplets of a few picoliters (approximately 5 pl). A single pulse waveform at 10 kHz jetting frequency was used for deposition, obtaining satellites-free spherical droplets with speeds higher than 5 m/s in the volume range of approximately few picoliters by setting a jetting voltage of 14 Volts.

### 2.3. Protein Deposition

**Figure 1:**
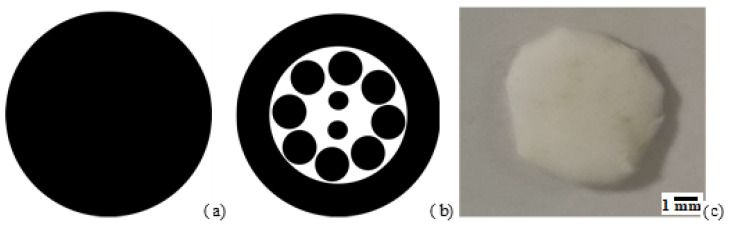
Printing patterns; pattern 1 (**a**) and pattern 2 (**b**). Photo of the scaffold post printing (**c**).

The BMP-2 protein solution was printed at room temperature (26–27 °C) by using a DMP-2800 Dimatix Materials Printer (FUJIFILM Dimatix, Inc., Santa Clara, CA, USA) at 40% relative humidity. This instrument was equipped with user fillable piezo-driven inkjet print cartridges, each with 16 nozzles 254 µm spaced 21.5 µm (10 pL) in diameter. The ejection of the droplet at the nozzles was executed by single pulse waveforms (i.e., the voltage vs. time signal given as input to the piezoelectric actuator) at jetting voltages in the range 15–16 V (frequency: 2 kHz) and constant drop spacing 5 µm. Such waveform has been previously used for protein solution in aqueous solutions (https://pubs.rsc.org/en/content/articlelanding/2016/lc/c6lc01072e) (accessed on 22 July 2024), allowing for aqueous ink deposition avoiding satellites or protein denaturation during dispensing. The deposition of BMP-2 protein ink on the scaffold was carried out by a series of layers, see below for the details, to almost fully print the BMP-2 ink (2 mL) loaded in the cartridge. Importantly, the design of the pattern was optimized to almost entirely print the protein ink in the scaffold, following a precise concentration gradient from the outside to the inside of the scaffold, for facilitating cells attach from the fractured bone, as shown in Figure 1.

To do so, the printing protocol was executed in two layers. In particular, pattern 1 (repeated 30 layers, Figure 1a), uniformly distributed proteins inks on the scaffold; pattern 2 (repeated 107 layers, Figure 1b) effectively produced the desired concentration gradient on the scaffold. For both layers, the droplet-to-droplet spacing was set to 5 microns, in order to increase the droplets density on the scaffold. A photo of the scaffold after the printing protocol is presented in Figure 1c. It is possible to note that the scaffold has some yellowish stripes, which could be a likely result of the protein ink printing and water evaporation on the scaffold.

### 2.4. Study Population

After exploring alternatives to animal model testing in bone scaffold research in the literature and taking into account the guidance given by the European Union Reference Laboratory for alternatives to animal testing (EURL-ECVAM) on alternative methods and acceptable approaches, the animal model (rabbit) was used. Procedures involving animals were carried out in accordance with the Italian Legislative Decree N° 26/2014 and the European Directive 2010/63/EU. The animals were housed and tested at the Istituto Zooprofilattico Sperimentale della Sicilia ‘A. Mirri’ with ministerial authorisation: 14/2015-UT. The number of animals used for this project is reduced to the minimum compatible with the verification of the scientific objectives, as required by the current legislation (Legislative Decree 26/2014). The present in vivo study was conducted on 6 male New Zealand white rabbits from the company Harlan Laboratories srl Zona Industriale Azzida, 57 33049-San Pietro al Natisone (UD), with an average body weight of 4.85 kg (range: 3.5–6 kg), 4–6 months old. Animals were housed in polypropylene cage and kept in controlled temperature (22 ± 2 °C), humidity (50–55%) and light (12 h light/dark cycle). Animals had access to food and water ad libitum. The rabbits were randomly divided into 3 groups of 2 individuals each and left to acclimatize for at least 2 days before the experiments. The groups were divided as follows. The control group was that of the rabbits whose defect was left to heal spontaneously. Group A was that of the rabbits whose defect was treated with the printed protein scaffold. Group B was that of the rabbits whose defect was treated with the scaffold with impregnated protein. One rabbit from each group was assigned to the 3-month follow-up and one to the 6-month follow-up. Then at each follow-up one rabbit per group was studied: control, printed protein (group A) and impregnated protein (group B).

### 2.5. Surgical Procedure

**Figure 2:**
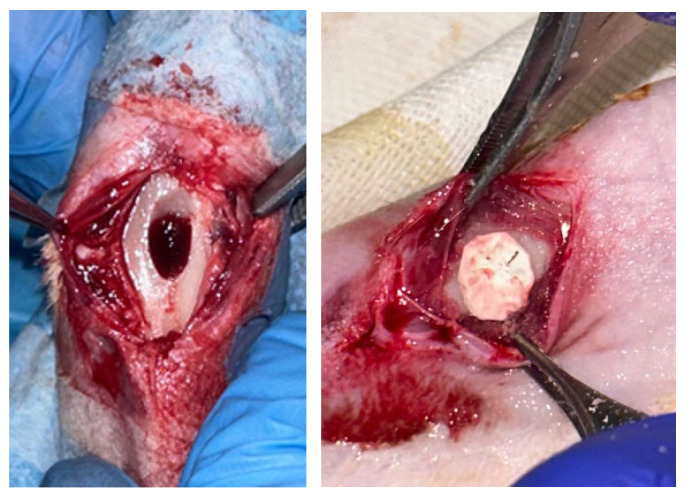
Application of the scaffold on the tibial defect.

The experimental procedure was carried out under general anesthesia and with the administration of analgesics and antibiotic therapy so as not to induce any pain, suffering or stress to the animal. Animals were induced to anesthetic depth with inhaled isoflurane at 2% and then anaesthetized with intramuscular (i.m.) injection of Zoletil(r) (tiletamine/zolazepam; 10 mg/kg) and Domitor(r) (medetomidine hydrochloride; 0.5 mg/kg). In a sterile field, after shaving and disinfecting the skin with iodine solution, a full-thickness incision was made along the frontal aspect of the proximal tibia. Retracting the skin flap and the periosteal sheet, an approximately 8 mm circular defect was created on the frontal aspect of the cortical bone of the tibia using a high-precision surgical drill (hand drill). Then a sterile spatula was used to create a cavity of approximately 5–6 mm. After removing the correct amount of bone tissue, the defect is washed with saline solution. All procedures were performed under constant saline irrigation to avoid friction damage to the bone. Then, the 8 mm scaffold was placed in the bone defect exerting a minimal pressure making the scaffold stable and covering the defect (Figure 2).

No suturing of the periosteum is performed. The skin is sutured with 3-0 silk and disinfected with iodine solution (Betadine).

I.m. atipamezole (Antisedan) (300 μg/kg) was used in order to awaken all rabbits. Carprofen (5 mg/kg) and Enrofloxacin (5 mg/kg) were daily administered for 1 week to each rabbit. After the procedure, each animal was assigned with an identification number and housed one per cage. They were monitored on a daily basis for infection, self-mutilation, and signs of distress.

### 2.6. Histology and Immunohistochemical Evaluation

**Figure 3:**
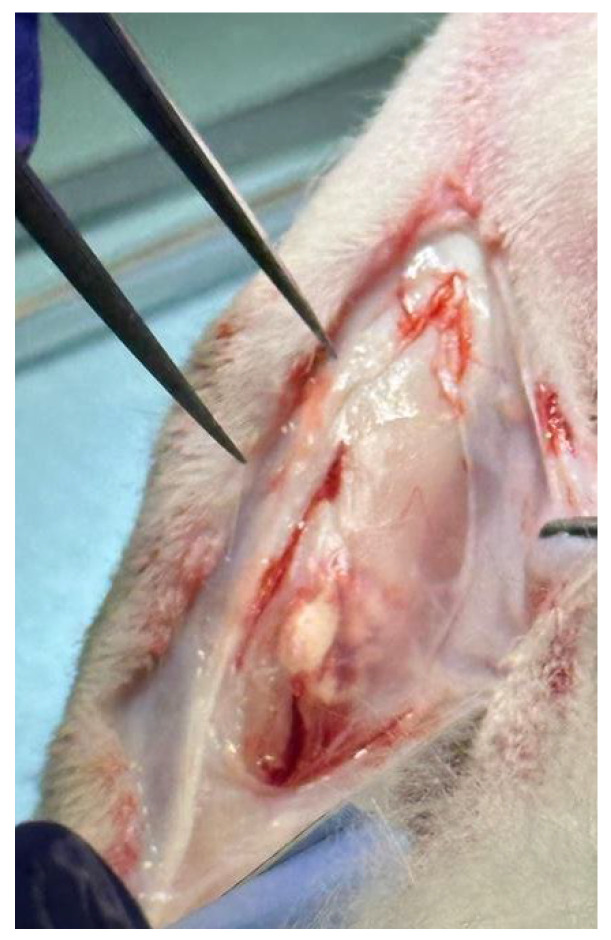
Surgical procedure for bone tissue collection.

At the follow-ups, the subjects were euthanised with Tranax (1 mL) intracardiacally. After shaving, an incision on the anterior aspect of the tibia was made to expose the bone (Figure 3). Using a high-precision surgical burr, the bone was harvested.

The histological examination was then conducted using the following procedures. Specimens fixed and preserved in 10% buffered formalin, were decalcified using Na EDTA (10% *w*/*v*, pH 7.2), prior to histological analysis. In order to perform histological examination, 4 μm thick sections were obtained by formalin-fixed paraffin-embedded tissue that were set on slides treated with silane (3-aminopropyl-trieossi-silane) in order to avoid section detachment, during staining. The preparations obtained were dried overnight in an oven at 37 °C, followed by dewaxing by xylene for 20 min. After a descending alcohol series (100%, 95%, 75% and 50%), slides were washed in distilled water and then stained with Haematoxylin and Eosin (HE). This was followed by the ascending scale of alcohols (50%, 75%, 95% and 100%) and clarification in xylene. After this phase, the slides were mounted in acrylic mounting medium (Eukitt^®^, O. Kindler GmbH, Herzogenaurach, Germany). Three microscope images were obtained with microscopy from three random areas for the sample and then evaluated to measure the newly formed bone area in different group (control, printed protein and impregnated protein scaffold). Morphology analysis was performed through a Leica DMLB microscope connected with a Nikon camera and analyzed using digital image analysis (Nikon NIS Br, Nikon Instruments Europe BV, Amsterdam, The Netherlands). Immunohistochemical analysis was performed for CD56 and SATB-2 (both markers of osteoblastic lineage-committed cells) [13]. In all treated and control samples, a semi-quantitative count was performed in an area corresponding to 3 contiguous high-power fields (HPF, ×400) using immunohistochemical staining for CD56 to assess osteoblast density (Novocastra™ Liquid Mouse Monoclonal Antibody CD56, NCAM, London, UK). Based on the values obtained, the statistical average of scaffold-treated and control samples was calculated. For SATB-2 immunoistochemical staining, a SATB-2 (clone SATBA4B10) mouse monoclonal antibody (Santa Cruz Biotechnology, Dallas, TX, USA) was used. Staining procedures were carried out using a BOND-III Fully Automated IHC and ISH Staining System (Leica Biosystems, Buccinasco, Italy), following the standard procedure.

### 2.7. Statistical Analysis

A descriptive analysis was conducted to investigate the variations between the study group and the control group.

## 3. Results

### 3.1. Scaffold Characterization

**Figure 4:**
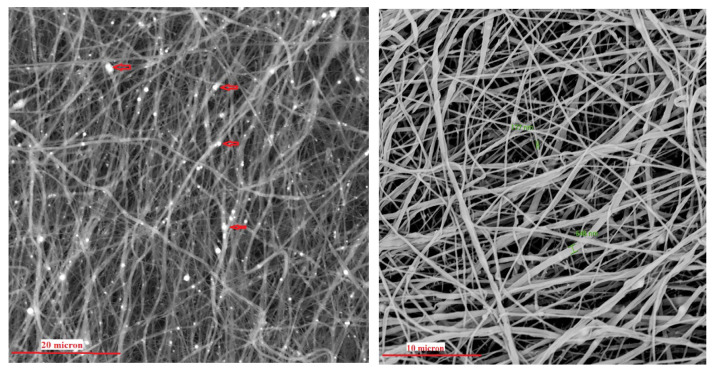
SEM image of electrospun PBS scaffold at 4000× (**left**) and 8000× (**right**) magnification. Red arrows of image at left indicate hydroxyapatite nanoparticles.

**Figure 5:**
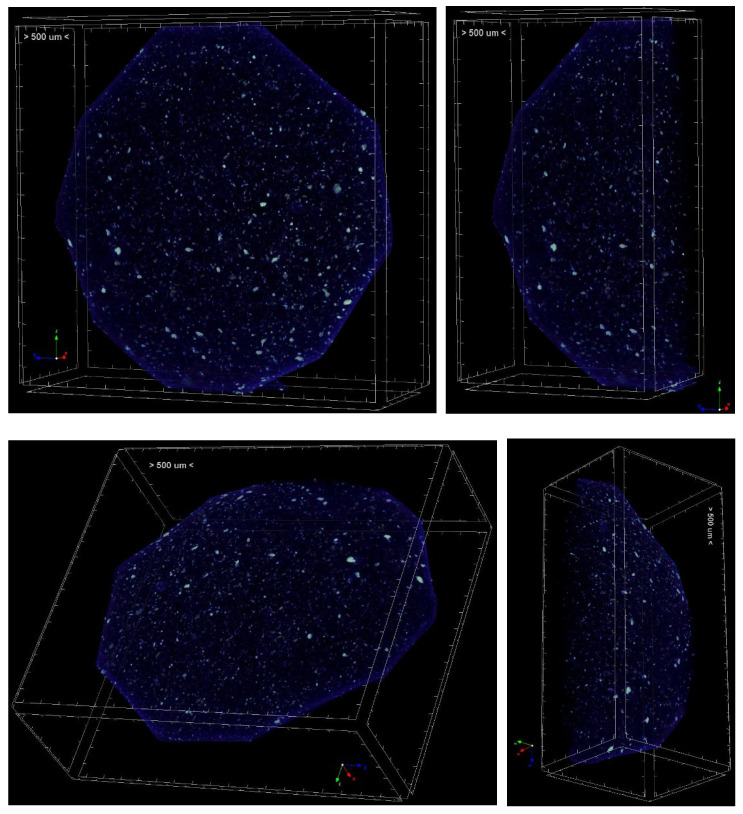
MicroCT reconstruction of PBS scaffold. White spots are hydroxyapatite nanoparticles.

**Figure 6:**
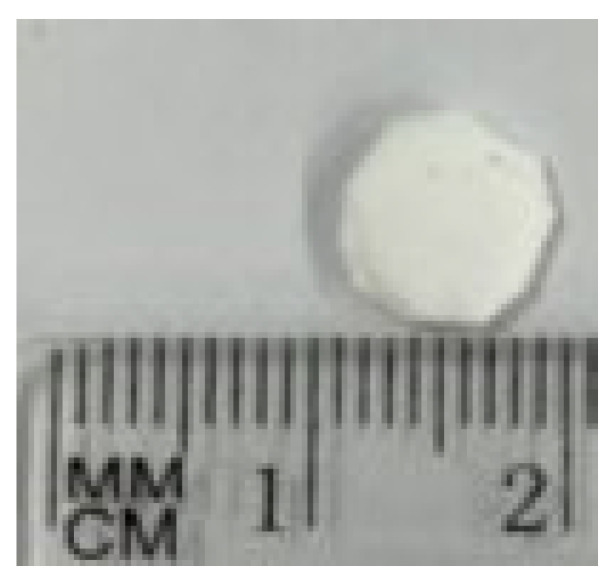
Photo of the 8 mm diameter PBS circular scaffold.

The morphology of the scaffold appears as a sheet of intertwined fibers, with a diameter of the fibers varies from a minimum of 150 nm to a maximum of 650 nm (Figure 4). Hydroxyapatite nanoparticles are also visible in the SEM image and computed Xray-microtomography (microCT). The scaffold has an average thickness of about 2.5 mm (measured by microCT, Figure 5) and sufficient rigidity for handling during implantation. For the other characterization, a specific experimental set-up was used. A manually cut 8 mm diameter circular scaffold was realized, customized to be implanted in rabbit bone defect (Figure 6).

### 3.2. Histological Analysis

**Figure 7:**
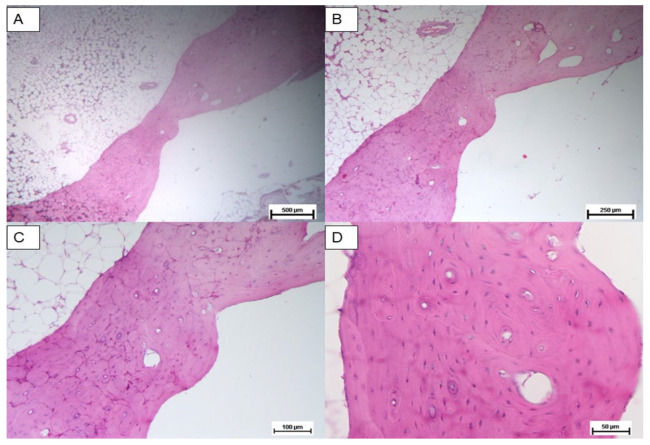
Control group: Hematoxylin and eosin stain of untreated bone defect; tibial defect (white area). Magnification: (**A**) 2.5×, (**B**) 5×, (**C**) 10×, (**D**) 20×.

**Figure 8:**
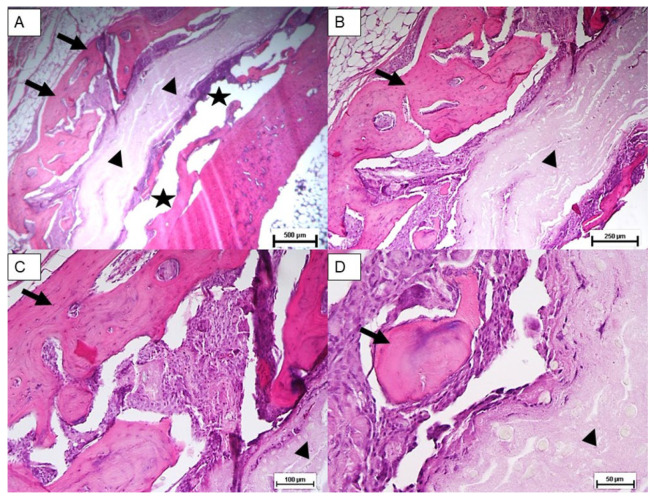
Group A: Hematoxylin and eosin stain of treated bone defect with the printed protein scaffold. New bone area (Arrow); scaffold (arrowhead); tibial defect (star). Magnification: (**A**) 2.5×, (**B**) 5×, (**C**) 10×, (**D**) 20×.

**Figure 9:**
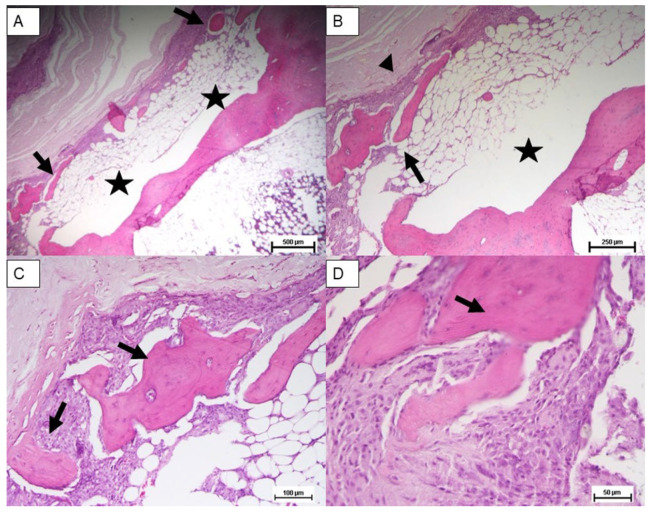
Group B: Hematoxylin and eosin stain of treated bone defect with the impregnated protein scaffold. New bone area (Arrow); scaffold (arrowhead); tibial defect (star). Magnification: (**A**) 2.5×, (**B**) 5×, (**C**) 10×, (**D**) 20×.

**Figure 10:**
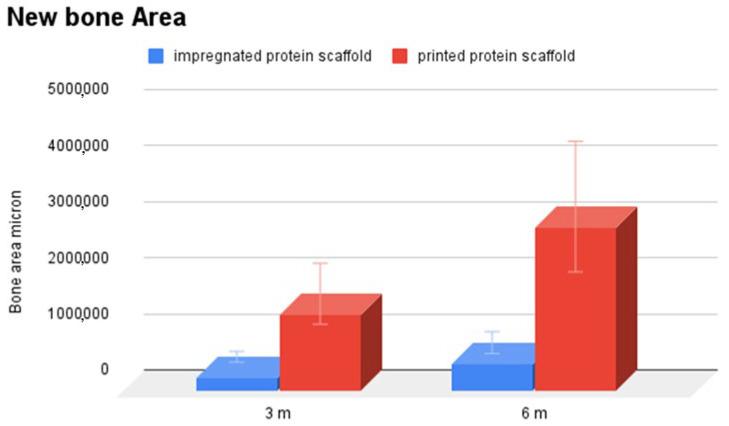
Comparison of newly formed bone tissue (micron^2^) at 3- and 6-months post implant of printed protein scaffold (group A) and impregnated protein scaffold (group B).

Histological analysis was carried out three months post implantation. The control group sample (Figure 7A–C) analyzed at 24 weeks showed only scant areas of newly formed bone tissue (not always observable) with only partial filling of the tibial defect. The tibial defect treated with the printed protein scaffold (Group A) highlighted not only areas of osteonecrosis with thinned and poorly mineralised bone lamellae, but also multiple areas of osteosynthesis with evidence of immature bone tissue in formation and numerous activated osteoblasts (Figure 8A–C); while samples of group B, whose defect was treated with the scaffold with impregnated protein, showed only a limited calcification of the scaffold (Figure 9A–C). The presence of amorphous material, at the tibial defect, was still evident; because, the scaffold, at this stage, is not completely degraded. Anyway, by using a digital image analysis software, a quantization of the areas of newly formed bone tissue was carried out. The results, shown in the graph of Figure 10, revealed a significant improvement of new bone formed area in the animal treated with the printed protein scaffold respect impregnated protein scaffold. At this stage, we certainly cannot confirm that the activity of the protein is higher if loaded with the printing process, but we can certainly assume that the latter guarantees a correct deposition of the protein on the entire surface of the scaffold and optimizes its surface distribution.

### 3.3. Immunohistochemical Analysis

**Figure 11:**
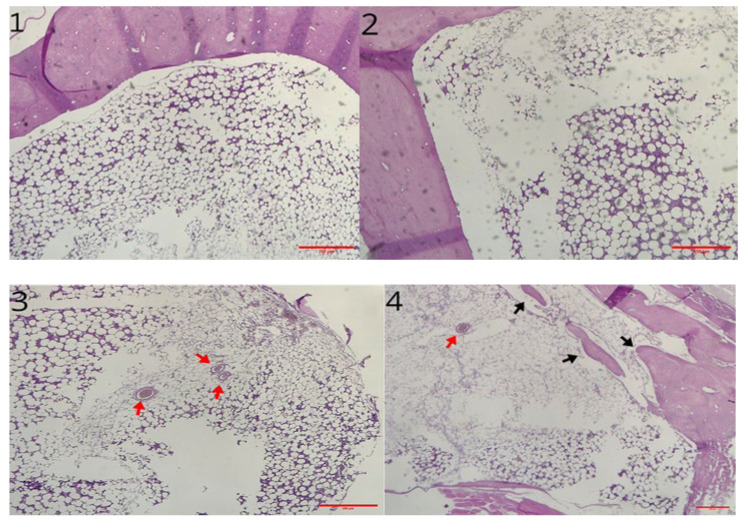
1, 2: Hematoxylin and eosin stain of control bone defect 3 months post surgery (magnification 4×). 3, 4: Hematoxylin and eosin stain of treated groups, 6 months post implant (magnification 4×). 3 was treated with printed scaffold (group A); 4 was treated with impregnated scaffold (red arrows mark areas of intramedullary neoangiogenesis; black arrows mark newly formed bone).

**Figure 12:**
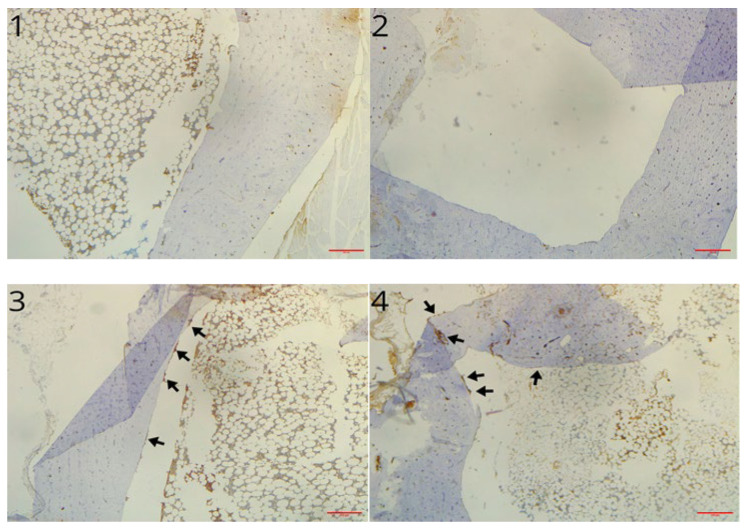
1, 2: CD56 immunochemical stain of control group bone defect 6 months post surgery (magnification 4×). 3, 4: CD56 immunochemical stain of treated bone defect 3 (3) and 6 (4) months post implant (magnification 4×); arrows indicate CD56+ osteoblasts.

The sample analysed at 3 months showed microscopically two healed defects in both the control and the scaffold-treated defect. In each case there was a slight reduction in parietal thickness. Areas of bone remodeling are evident in the untreated defect (control group), with areas of osteonecrosis. The medullary cavities showed both adipose and haemopoietic marrow. Differently, samples of printed protein scaffold (group A) showed areas of osteosynthesis with evidence of activated osteoblasts and numerous areas of intramedullary neoangiogenesis. No inflammatory infiltrates of any kind are evident (Figure 11). Additionally, immunohistochemical staining for CD56 showed a marked increase in the number of osteoblasts in the scaffold-treated samples compared to the untreated ones (Figure 12). Immunochemical staining for SATB2 (a fairly novel stain specific for osteoblastic lineage-committed cells) showed comparable results (images not reported).

### 3.4. Macroscopic Observation

**Figure 13:**
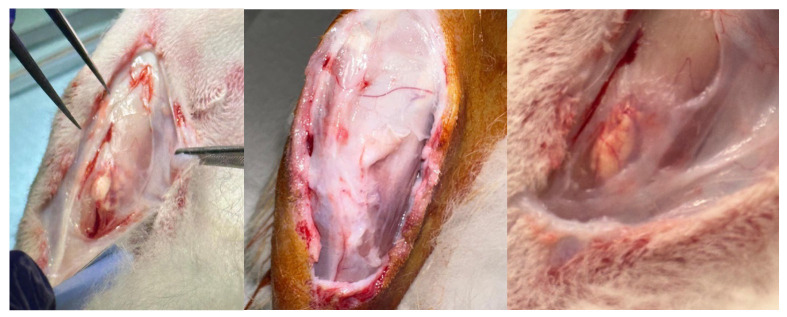
Photo of the harvest of the specimens before incision of the periosteal tissue. The absence of alteration of the bone profile in the control group can be seen in the picture on the left. The formation of hypertrophic callus or heterotopic bone can be observed in the study groups, Group A in the centre and Group B on the right.

Upon excision of the specimens, macroscopic observation of the samples shows a significant difference in the bone profile (Figure 13). The formation of hypertrophic callus or heterotopic bone is to be identified as a complication. However, the animals do not show any health problems in general or related to the implant area. The periosteal tissue, still present in the photos in Figure 13, also shows no alteration. Furthermore, the histological examination shows no significant alterations.

## 4. Discussion

For the first time, in a rabbit animal model, this study demonstrates that a microfibrillar PBS scaffold is effective in improving bone regeneration in critical bone defects on the tibia, a long bone. Furthermore, a scaffold is created in PBS with hydroxyapatite and ciprofloxacin and engineered with BMP-2. The employ of inkjet printing BMP-2 in mild conditions shown in this work into the PBS scaffold allows avoiding extrusion-based 3D printing which could in principle affect its bioactivity due to the printing temperature (https://link.springer.com/article/10.1007/s10439-021-02736-9) (accessed on 2 May 2025).

Therefore, this study demonstrates the enormous potential of PBS scaffolds applied to bone tissue. The device designed and studied in this project also demonstrates easy applicability as a surgical device, without intra- or post-operative complications. This study confirms the biocompatibility and biodegradability of PBS-based 3D scaffolds, and although on a small number of animals, it was possible to define the most suitable scaffold engineering model of growth factor and antibiotic. The difference in the macroscopic bone profile of the Group A and B samples, compared to the control group, shown in Figure 13, is objective.

The study group, regardless of the protein addition method, shows a reaction such as a hypertrophic callus or heterotopic bone formation. As described in the literature, this is attributable to the use of supraphysiological doses of BMP-2 [23]. Clinical studies have shown no cause for concern of oncological transformation although at this preclinical stage it is difficult to determine the clinical significance of this effect in humans. However, histological analysis shows increased bone regeneration in the study group samples. This represents the first and most important result of the study. Implanting the scaffold on the bone defect offers an advantage over spontaneous bone healing. In addition, the scaffold with printed protein (Group A) shows significantly more areas of osteosynthesis and activated osteoblasts than Group B. The quantification of new bone tissue, which tends to fill the defect created, was carried out for descriptive purposes only, given the small number of samples, which also show a high variability. In any case, the printed scaffold showed a greater tendency to produce new bone tissue than the impregnated one.

The immunohistochemical analysis performed at 6 months based on CD56 and SATB2 also qualitatively demonstrates the advantage of the scaffold over spontaneous bone healing. Thus, at both follow-ups, no inflammatory infiltrates are present and the scaffold shows a definite advantage over spontaneous bone healing. In addition, the printed protein scaffold proves to be the device with the greatest potential.

The search for a scaffold with osteogenic and osteoinductive properties finds considerable attention in the literature. Other studies show similar results to those described in this study, although it is almost impossible to compare them in terms of results. A similarity in study design can be found in the work that Teotia et al. used to validate the scaffold properties. They studied their scaffolds, consisting of a 3D-printed porous composite structure with resins and osteoinductive growth factors, on both flat bone (skull theca) and long bone (tibia) [9]. In 2018, Preethi Soundarya et al. [5] provided an overview of different fabrication techniques for the production of scaffolds, but concluded that an ideal production method for a scaffold has not yet been determined. Therefore, considering the results of the current study, the authors believe that the microfibrillar PBS scaffold produced by electrospinning may be a viable alternative to those proposed in the current literature. Furthermore, the biomechanical characteristics of the scaffold produced in this study allow its easy surgical applicability even on long bones. This feature is of fundamental importance in a study with translational objectives. The main limitation of the study is the small number of samples. This limits the possibility of statistical analysis and also the weight of qualitative evidence in the study. Unfortunately, the limits are imposed by legislative restrictions. A further limitation may be the not easy reproducibility of the study in all its phases. The authors believe that the methodology chosen for the sample analysis is sufficient to achieve the objective of this study. Future studies will require a more extensive analysis in terms of numbers and methods to validate and quantify the benefits of the printed scaffold.

## 5. Conclusions

In conclusion, this study demonstrates, for the first time, that a microfibrillar PBS-hydroxyapatite-ciprofloxacin scaffold with BMP-2 can be created and is effective in improving bone regeneration in tibia critical bone defect in a rabbit animal model. The absence of adverse events demonstrates the scaffold’s biocompatibility and biodegradability properties. Furthermore, the scaffold made with the printed protein proves to be the most effective in enhancing osteoinductive activity compared to the other model and the control group left to heal spontaneously. Finally, from a surgical point of view, the device designed and studied in this project demonstrates optimal physical characteristics for easy applicability during surgery and no intra- or post-operative complications.

## Data Availability

Data will be available upon request to corresponding author.
